# A retrospective study on the clinical, radiological, and microbiological characteristics of empyema thoracis in Qatar

**DOI:** 10.5339/qmj.2024.qitc.2

**Published:** 2024-03-25

**Authors:** Theeb Osama Sulaiman, Mousa Hussein, MOHD Yaseen, Mansoor Hameed, Abdelnasser Elzouki, Merlin Thomas

**Affiliations:** 1 Department of Chest, Hamad General Hospital, Doha, Qatar Email: tsulaiman@hamad.qa; 2 Department of Clinical Medicine, Qatar University, Doha, Qatar; 3 Department of medicine, Hamad General Hospital, Doha, Qatar; 4 Department of Clinical Medicine, Weill Cornell Medicine, Doha, Qatar

**Keywords:** Empyema, Qatar, Streptococcus

## Introduction

An empyema is a collection of pus in the pleural space, which leads to various complications. Retrospective series report a mortality of approximately 15% in empyema patients who are admitted to the hospital.^1,2^

The main objective of this study is to describe the epidemiology, clinical characteristics, management, and outcomes of patients with empyema in Qatar.

## Methods

Using electronic records from Hamad Medical Corporation hospitals, the national referral center for Qatar, we identified patients aged ≥18 years diagnosed with empyema between January 1, 2015, and December 31, 2019. Data on demographic information, comorbidities, laboratory results, imaging findings, microbiology, antimicrobial therapy management, and complications were collected.

## Results

Seventy-five empyema cases were reviewed, predominantly males (89.3%) with a mean age of 44 years. Diabetes mellitus (30.7%) and a history of malignancy (20%) were prevalent comorbidities. Table 1 shows the baseline characteristics of the patients.

Clinical symptoms of the cohort included shortness of breath (60.3%), chest pain (54.7%), and fever (52.7%). The pleural fluid with turbid color was universally exudative (55.4%). Streptococcus (38.7%) and Gram-negative bacteria (29.3%) were predominant. Table 2 shows the characteristics and microbiology of the pleural fluid.

Antibiotics were administered to all patients, with chest tube insertion being the primary intervention in 74.6%. Video-assisted thoracoscopic surgery (VATS) and medial thoracoscopy were used in 24% and 1.3%, respectively. Of the patients who were managed by chest tube insertion, 7.14% required VATS. Of the patients, 45.3% achieved complete resolution, whereas 34.6% did not have follow-up imaging. Complications were minimal in 1.3%, but mortality was noted in 18.6%.

## Conclusion

This study highlights that streptococci are the predominant etiological agent in empyema cases in Qatar, with diabetes mellitus being the major comorbidity. Early antibiotic administration and chest tube insertion emerged as crucial components of effective management, often obviating the need for surgical interventions, although mortality remains a major concern.

## Conflict of Interest

I have no conflict of interest.

## Figures and Tables

**Table 1.** Baseline characteristics of the patients.

**Clinical characteristic**	**Number of patients, N (%)**
Mean age, years, mean ± SD	44 ± 15.6
Gender	
Males	67 (89.3%)
Smoking	
Non-smoker	31 (49.2%)
Ex-smoker	14 (22.2%)
Alcohol	
Alcohol consumer	6 (9.5%)
Ex-alcohol consumer	4 (6.3%)
**Comorbidities**	
Diabetes mellitus	23 (30.7%)
Hypertension	19 (25.3%)
Neoplastic disease	15 (20%)
Prior history of thoracic procedure	14 (18.7%)
Prior history of pneumonia	13 (17.3%)
History of other known medical conditions*	23 (30.7%)

* Prior history of pleural effusion/empyema, 7 (9.3%); prior history of any relevant surgery or illness, 5 (6.7%); chronic kidney disease, 3 (4%); history of cerebrovascular disease, 3 (4%); chronic liver disease, 2 (2.7%); history of asthma, 1 (1.3%); history of COPD, 1 (1.3%); chronic heart failure, 1 (1.3%).

**Table 2.** Characteristics and microbiology of the pleural fluid.

**Parameter**	**Number of patients, N (%)**
**Appearance**	
Turbid	36 (55.4%)
Bloody	11 (16.9%)
Milky	6 (9.2%)
Straw	7 (10.9%)
Brown	4 (6.2%)
**Microbiology**	
Streptococcus	29 (38.7%)
Staphylococcus	21 (28%)
Enterococcus	6 (8%)
Gram-negative bacteria	22 (29.3%)
Pseudomonas	6 (27.2%)
Klebsiella pneumonia	5 (22.7%)
Enterobacter cloacae	3 (13.6%)
Escherichia coli	3 (13.6%)
Acinetobacter baumannii	3 (13.6%)
Candida albicans	2 (2.6%)
